# Population Explosion in the Yellow-Spined Bamboo Locust *Ceracris kiangsu* and Inferences for the Impact of Human Activity

**DOI:** 10.1371/journal.pone.0089873

**Published:** 2014-03-06

**Authors:** Zhou Fan, Guo-Fang Jiang, Yu-Xiang Liu, Qi-Xin He, Benjamin Blanchard

**Affiliations:** 1 Jiangsu Key Laboratory for Biodiversity and Biotechnology, College of Life Sciences, Nanjing Normal University, Nanjing, China; 2 Department of Ecology and Evolutionary Biology, University of Michigan, Ann Arbor, Michigan, United States of America; University of Florence, Italy

## Abstract

Geographic distance and geographical barriers likely play a considerable role in structuring genetic variation in species, although some migratory species may have less phylogeographic structure on a smaller spatial scale. Here, genetic diversity and the phylogenetic structure among geographical populations of the yellow-spined bamboo locust, *Ceracris kiangsu*, were examined with 16S rDNA and amplified fragment length polymorphisms (AFLPs). In this study, no conspicuous phylogeographical structure was discovered from either Maximum parsimony (MP) and Neighbor-joining (NJ) phylogenetic analyses. The effect of geographical isolation was not conspicuous on a large spatial scale.At smaller spatial scales local diversity of some populations within mountainous areas were detected using Nei's genetic distance and AMOVA. There is a high level of genetic diversity and a low genetic differentiation among populations in the *C. kiangsu* of South and Southeast China. Our analyses indicate that *C. kiangsu* is a monophyletic group. Our results also support the hypothesis that the *C. kiangsu* population is in a primary differentiation stage. Given the mismatch distribution, it is likely that a population expansion in *C. kiangsu* occurred about 0.242 Ma during the Quaternary interglaciation. Based on historical reports, we conjecture that human activities had significant impacts on the *C. kiangsu* gene flow.

## Introduction

Highly migratory species are usually expected to have minimal population substructure over their distributional ranges because strong gene flow can counteract the isolating effects of geographical distance and physical barriers [1]. The current distribution of genetic variation within a species is a product of its demographic history as well as the interaction between mutation, genetic drift and gene flow. It is notoriously difficult to distinguish historical effects, like climate change or geological accident, from shorter-term consequences of dispersal patterns and limited population size, but the distinction is important [2]. It is the imprint of historical changes on the distribution and abundance of organisms left in current population structure that underpins phylogeography [3]. Often these spatial genetic patterns are on a much larger scale than the correlations generated by dispersal of individuals in stable populations [2].

The Quaternary glaciation period played an important role in shaping the history of global fauna and flora. In the past few decades, glacial cycles have been considered one of the most important factors in shifting population genetic structure and promoting floral and faunal diversification [4]–[10]. Because of climate changes species range of temperate organism is contracted during the glacial periods and is expanded during the interglacial periods [11]. [12]. Glacial cycles acting on genetic diversity of grasshoppers have been well studied in Europe and North America [13]–[16]. The genetic legacy of Pleistocene influence remains poorly understood for Southeast Asia, where glaciation was not synchronous with the Northern Hemisphere ice sheet maxima [2], [16]–[17]. China, especially south of China (e.g. Yunnan province), provided some key refugia for many relict rare species during the Pleistocene glaciation, due to its complex topography and numerous large mountains spanning from west to east [18], [19]. Zhang et al. [20] studied the population structure of the migratory locust *Locusta migratoria*, an infamous pest insect with exceptional migratory ability, and discovered its minimal population substructure. However, most other grasshoppers do not have excellent ability of flying to undergo such rapid climatic change. For instance, limitations due to resources like food may have prevented large-scale colonization by insects [12]. Another example in China is that the agriculture pest *Oxya hyla* intricate was separated into three deep monophyletic clades with no shared haplotype in a study by Li et al. [10]. They suggested that climatic oscillations during glacial periods in the Quaternary caused a complete demographic expansion pattern of *O. hyla* intricate populations, and that past geological events and climatic fluctuations are the most important factor in shaping the population genetic diversity and structure of the species.

The yellow-spined bamboo locust, *Ceracris kiangsu* Chai, is one of the most severe bamboo pests endemic to China [21]. It is mainly distributed in South China and is characterized by its behavior of feeding in large groups on the leaves of bamboo plants during all of its life stages [21]–[23]. Because of this feeding habit, their distribution range is more restricted than *L. migratoria* [20] and *O. hyla* intricate [10]. Scholars have long explored the morphological taxonomy, pesticide toxicology, physiology and development of *C. kiangsu* [24]–[29]. However, to our knowledge, there is only one published report of genetic studies on bamboo locust [30].

Here, we use Amplified fragment length polymorphisms (AFLPs) and 16S rDNA datasets to obtain more information about the population histories of *C. kiangsu* and infer demographic processes. AFLPs have been shown to provide phylogenetic resolution among recently and rapidly radiating groups in which sequence data have failed [31]–[33]. The increased resolution of AFLPs is associated with their nuclear genome-wide distribution, which overcomes problems associated with locus-specific effects, and the large number of polymorphisms that can be easily characterized. Recent population genetic analyses of multiple *Heliconius* species based on AFLPs revealed pronounced genetic structure in both *H. erato* and *H. melpomeneover* at small spatial scales [34], [35], suggesting that AFLPs should be effective at distinguishing closely related and geographically proximate races in each species. Together, mtDNA and nuclear markers can provide complementary information about population structure and evolutionary patterns of *C. kiangsu*.

In this study, we compare the resolution of both marker types and address the following questions: (1) In the context of the unregularly phylogeographic structure of *C. kiangsu*, how long has geographical distance influenced the gene flow between different local populations? (2) Does geographical isolation affect the genetic structure of *C. kiangsu* in modern times? (3) If this species experienced a genetic bottleneck and/or a population explosion, did Pleistocene climate cycles play a significant role in shaping the history of *C. kiangsu*, similar to *O. hyla* intricata or other organisms? Additionally, we also estimate the minimum age of this bamboo locust based on pairwise mtDNA sequence divergence. Our results illuminate the population structure in the bamboo locust *C. kiangsu* and provide essential insights for future work focused on the forest grasshopper's response to climate changes.

## Materials and Methods

### Ethics statement

This species of grasshopper in this study is a famous agricultural pest in China, need to control. Collections of samples were permitted by the authority of each forestry center (Forestry Bureau of Guangning County of Guangdong Province, Forestry Department of Hunan Province, Forestry Bureau of Taoyuan County of Hunan Province, Forestry Bureau of Hengyang City of Hunan province, Vegetation Protection Central Station of Guangxi Zhuang Autonomous Region, Forestry Bureau of Rongshui County of Guangxi Zhuang Autonomous Region, Forestry Bureau of Quzhou County of Zhejiang Province, Forestry Bureau of Jianou County of Fujian Province, Forestry Bureau of Guigang City of Jiangxi Province, Forestry Bureau of Shicheng County of Jiangxi Province, and Forestry Bureau of Pingxiang City of Jiangxi Province). For collection locations no specific permits were required for the described field studies because sample collection did not involve endangered or protected plant species or privately-owned locations.

### Sample collection and DNA extraction

**Figure 1:**
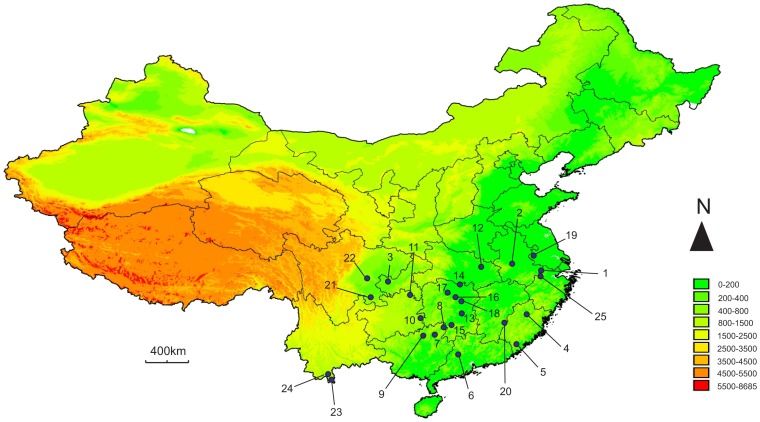
Locations of C. kiangsu populations sampled for AFLP and mtDNA analyses.

A total of 393 individuals of *C. kiangsu* were collected from 24 locations in the field covering nearly all of the species' distribution range between 2007 and 2011 (Figure 1; Table 1). A total of 25 populations were included in our foregoing analyses. Considering the migration ability of *C. kiangsu*, most populations were collected within regions around 200 km apart, except Menglun and Mengla (90 km). Furthermore, there is a special bamboo species, *Bambusa textiles* McClure, in Guangning County, while the species in all of the other locations is the bamboo *Phyllostachys heterocycla* (Carr.) and/or others. Two individuals of the green-spined bamboo locust *C. nigricornis* collected in Shuangpai County were used as outgroups. All samples were stored in 100% ethanol upon capture.

**Table 1:** Sampling site, geographical coordinates, elevation (metres), sample size (*Nind*) of sampled *Ceracris kiangsu* populations, and sequence information of each geographical population calculated from DnaSP software.

NO	Sampling site	Latitude	Longtitude	Elevation	*N_ind_*	*S*	*h*	*H_d_*	Variance of H_d_	St.Dev	*Pi*	Sampling variance	St.Dev
**1**	**Guangde, Anhui**	30°47′19″N	119°28′51″E	216	15	3	3	0.448	0.018	0.134	0.002	0.000	0.001
**2**	**Shucheng, Anhui**	31°22′05″N	116°59′13″E	42	15	49	3	0.362	0.021	0.145	0.014	0.000	0.011
**3**	**Jinyunshan Chongqing**	29°50′14″N	106°23′46″E	600	25	0	1	0.000	0.000	0.000	0.000	0.000	0.000
**4**	**Jianou, Fujian**	27°02′08″N	118°14′14″E	194	14	24	3	0.275	0.022	0.148	0.013	0.000	0.007
**5**	**Nanjing, Fujian**	24°29′18″N	117°21′01″E	345	2	0	1	0.000	0.000	0.000	0.000	0.000	0.000
**6**	**Guangning, Guangdong**	23°36′17″N	112°23′18″E	52	34	21	3	0.266	0.008	0.092	0.010	0.000	0.004
**7**	**Guilin, Guangxi**	25°18′25″N	110°23′40″E	277	10	0	1	0.000	0.000	0.000	0.000	0.000	0.000
**8**	**Quanzhou, Guangxi**	25°55′43″N	111°09′44″E	286	20	1	2	0.100	0.008	0.088	0.000	0.000	0.000
**9**	**Rongan, Guangxi**	25°12′29″N	109°23′45″E	160	18	9	2	0.111	0.009	0.096	0.002	0.000	0.002
**10**	**Jinping, Guizhou**	26°43′04″N	109°10′52″E	552	6	0	1	0.000	0.000	0.000	0.000	0.000	0.000
**11**	**Mayanghe, Guizhou**	28°41′47″N	108°16′16″E	662	2	0	1	0.000	0.000	0.000	0.000	0.000	0.000
**12**	**Wuhan, Hebei**	31°05′30″N	114°21′01″E	100	20	2	3	0.279	0.015	0.123	0.001	0.000	0.000
**13**	**Hengyang, Hunan**	27°07′01″N	112°41′36″E	140	4	0	1	0.000	0.000	0.000	0.000	0.000	0.000
**14**	**Huarong, Hunan**	29°35′23″N	112°32′17″E	32	20	2	3	0.563	0.004	0.063	0.001	0.000	0.000
**15**	**Shuangpai, Hunan**	26°06′30″N	111°49′28″E	460	18	24	2	0.111	0.009	0.096	0.006	0.000	0.005
**16**	**Taojiang, Hunan**	28°30′09″N	112°09′08″E	55	16	13	10	0.917	0.002	0.049	0.006	0.000	0.002
**17**	**Taoyuan, Hunan**	28°54′09″N	111°29′20″E	183	46	4	5	0.572	0.001	0.037	0.001	0.000	0.000
**18**	**Changsha, Hunan**	28°10′11″N	112°40′06″E	65	19	1	2	0.526	0.001	0.040	0.001	0.000	0.000
**19**	**Zijinshan, Jiangsu**	32°04′14″N	118°50′57″E	170	20	2	3	0.595	0.005	0.073	0.001	0.000	0.001
**20**	**Shicheng, Jiangxi**	26°19′35″N	116°20′36″E	408	19	0	1	0.000	0.000	0.000	0.000	0.000	0.000
**21**	**Changning, Sichuan**	28°29′57″N	104°55′58″E	309	19	19	3	0.368	0.0158	0.016	0.005	0.000	0.004
**22**	**Ziyang, Sichuan**	30°07′45″N	104°37′44″E	355	5	0	1	0.000	0.000	0.000	0.000	0.000	0.000
**23**	**Mengla, Yunnan**	21°29′18″N	101°33′23″E	771	5	68	5	1.000	0.016	0.126	0.073	0.000	0.014
**24**	**Menglun, Yunnan**	21°55′25″N	101°15′56″E	549	7	44	6	0.952	0.009	0.096	0.425	0.000	0.010
**25**	**Quzhou, Zhejiang**	30°19′03″N	119°25′57″E	191	14	1	2	0.143	0.014	0.119	0.003	0.000	0.000
	**Total**				393	122	33	0.436	0.001	0.029	0.060	0.000	0.001

*S*, number of polymorphic/indel/missing sites; *h*, number of haplotypes; *Hd*, haplotype diversity; sampling variance of *Hd*; St.Dev, standard deviation of haplotype diversity; *Pi*, nucleotide diversity, sampling variance of *Pi*, and standard deviation of *Pi*.

Genomic DNA was extracted from femur samples using Wizard® Genomic DNA Purification Kit (PROMEGA, USA) following the manufacturer instructions, and stored at −30°C until needed. All DNA extracts for AFLP were run on 1% agarose gels, and samples that did not have high concentrations of high molecular weight DNA or that appeared excessively sheared were not included in AFLP analysis [36]. Sample sizes used for AFLP analysis from collected populations ranged from 2 to 10, and all individuals were used in mitochondrial DNA analysis.

### Mitochondrial DNA

A 483 bp segment of the 16S rRNA gene was amplified in a subset of 393 individuals with primers given in Liu & Jiang (2004): LR-J-12887 (5′- CCGGTCTGAACTCAGATCACGT-3′) and LR-N-13398 (5′-CGCCTGTTT AACAAAAACAT-3′). PCR reactions were carried out in 50 µL volumes which included 1 µL template DNA (total genomic DNA diluted 1∶10 with water), 5 µL 10×buffer, 3 µL MgCl_2_, 4 µL dNTPs, 1 µL of each primer and 0.25 µL rTaq (TaKaRa Bio Inc,Dalian,China). PCR cycling conditions included an initial 5 min at 94°C, 30 cycles of 95°C for 30 sec, 56°C for 30 sec, 72°C for 1 min,plus a final extension at 72°C for 10 min.

PCR products were tested by electrophoresis on a 1% agarose gel, and all PCR products were sequenced on ABI-PRISM3730 with BigDyeterminator v3.1 (Shanghai Sangon Biotech Co., Ltd.). Alignments are available on request. To confirm the identity of the mtDNA sequences, the amino acid sequences were inferred using GeneDoc 2.6.02 based on the invertebrate mitochondrial code.

### AFLPs

We applied the original AFLP protocol of Vos et al. (1995) with a few modifications [37]. Those individuals with genomic DNA which has high consistency and purity quotients were used in this part. Because locust species have a larger genome than many other insects, we performed a longer enzyme digestion in order to obtain more polymorphic fragments. 400 ng genomic DNA was digested at 37°C with the enzymes 0.2 µL EocRI and 0.5 µL MseI (both Fermentas, with 2× Buffer R) for 3 h followed by 70°C for 15 min to ensure enzyme inactivation. EocRI/MseI adapters were ligated to the enzyme digestion product using T4-DNA-Ligase (FERMENTAS) at 20°C overnight. We used 16 primer combinations containing one selective base. Preselective amplification was conducted in a total volume of 30 µL including 3 µL of diluted retriction-ligation DNA, 3 µL 10× buffer, 2.4 µL MgCl_2_, 1.6 µL dNTPs, 0.4 µL rTaq (TAKARA) and 1 µL EocRI+N primer and 1 µL MseI+N primer, up to 30 µL with ddH_2_O. The thermal cycling parameters for preselective amplification were 2 min at 94°C, 30 cycles of 30 sec at 94°C, 1 min at 56°C, 1 min at 72°C, followed by 10 min at 72°C. The ligations were diluted 1∶20 and 2 µL of the diluted preselective amplification product was used in the selective amplification. Selective amplification was conducted in a total volume of 20 µL with 2 µL diluted preselective amplification product, 2 µL 10× buffer, 1.6 µL MgCl_2_, 1.6 µL dNTPs, 0.6 µL rTaq (TAKARA) and 1 µL EocRI+3 primer (labeled with FAM) and 1 µL MseI primer, up to 20 µL with ddH_2_O. Four primer combinations (E–AGG/M–CAG, E–AGG/M–CTT, E–AGC/M–CTC, E–AAG/M–CAG, where E is EocRI, M is MseI) were used in this step. Selective amplification products were visually measured on an ABI 3700 DNA analyzer (Shanghai Sangon Biotech Co., Ltd.).

Fragment data were analyzed with GeneMarker version 2.20 (Demo). We scored fragments of size 50–500 bp as present or absent. Minimum fragment signal intensity was initially used for all fragments. The signal intensity was measured as relative fluorescent units (RFU) of 500 or 1000 depending on the primer set [36].

### Data analysis

#### mtDNA

The mitochondrial DNA sequence data were assembled and corrected in SeqMan II version 5.08 (Lasergene, DNASTAR Inc., Madison, WI). We then carried out alignments in MEGA 5 [38] based on the ClustalW function. All the sequences were uploaded to NCBI (Accession nos: JQ799527-799883).

The number of haplotypes (h) as well as haplotype diversity (H_d_) and the number of variable sites were analyzed in DnaSP v5.10 [39] with aligned sequences. Other information about geographical populations included number of segregating sites (S), and nucleotide diversity (Pi). The sequence sets were used to calculate F_ST_ and the p value in the AMOVA function of ARLEQUIN v3.11 [40]. We also calculated indices about gene flow between geographical groups. In genetic structure test, we divided 25 population into 18 groups acording to geographical distance. Two selective neutrality tests of Tajima' D [41], [42] and Fu's F [43] were also performed in ARLEQUIN v3.11 [39], of which index below zero indicates that the species has experienced population dynamics, and a p value in both tests indicates significance. Sum of square deviation (SSD) and the Harpending's raggedness index (HRI) were also calculated in our analysis. Furthermore, a τ value was generated in mismatch analysis. The formula, τ = 2 ut was used to detect the population expansion time [44]. The nucleotide substitution rate in mitochondrial DNA was 2.3% per million years (MY) as suggested in Knowles et al. (2000) [45].

To represent phylogeographical structure among the haplotypes, a median-joining network was generated for all haplotypes using software NETWORK version 4.6.1.0 (Fluxus Technology Ltd.), by the Median Joining method [46]. Maximum parsimony (MP) and Neighbor-joining (NJ) phylogenetic analyses were used to identify major clades and evaluate the relationships among the haplotypes of the 16S. The TVM model (Transversional model) [47] selected by AIC in Modeltest 3.7 [48], [49] was used in 16S maximum parsimony analyses with PAUP* 4.0b10 [50]. Neighbor-joining (NJ) analyses were performed in MEGA5 using Kimura 2-parameter gamma model with 1000 bootstrapping replicates for 16S [51]. The Kimura's two-parameter gamma model corrects for multiple hits, and evolutionary rates among sites are modeled using a Gamma distribution.

#### AFLP analysis

Fragmentized information was exported as 0/1 popmatrix by GeneMarker and transformed in format in the AFLPDAT program [52], a collection of R functions which facilitates the handling of dominant genotypic data and can convert data from tables into input formats of several programs including ARLEQUIN, AFLP-SURV, STRUCTURE [53] POPGENE and others [52]. ARLEQUIN v3.11 was used in evaluating genetic variation among 25 geographic populations. Population pairwise F_ST_ (Pairwise genetic distance) and p value (the significance of F_ST_ value) with 1000 bootstrapping replicates were produced using AMOVA analysis in ARLEQUIN. And we also run a genetic structure test with the same groups we shown in *16S rRNA*.

Using AFLP-SURV [54], we estimated allelic frequencies by applying the Bayesian method with non-uniform prior distribution of allele frequencies [55]. When we use the Bayesian method, two important indexes were considered: sample size and the number of individuals lacking AFLP fragment. The frequency of the null (−) allele at each locus was computed as well as the distribution of allele frequencies based on the variation over loci frequencies. Using the 10000 bootstrapped matrices of Nei's genetic distance estimated in AFLP, a Neighbor-joining tree was constructed using the Neighbor and Consense programs in Phylip 3.6 [56].

The percentage of polymorphic loci (P), observed number of alleles (Na), effective number of alleles (Ne), gene diversity (He) [57], Shannon's Information index (I), and Nei's genetic distance [57] were calculated in POPGENE v1.31 [58] to examine the population genetic diversity and differentiation of *C. kiangsu*. POPGENE v1.31 [59] was also used to detect gene flow among different geographical populations.

To further investigate population structure, the assignment test and population structure examination were conducted with Bayesian assignment analysis implemented by the program STRUCTURE v2.3 [53]. STRUCTURE placed the 203 individuals into *K* subgroups that had distinctive allele frequencies without a priori population information, and a *K* index was set from 2 to 16. The output, Pr (*X|K*), was used as an indication of the number of groups [60], and each test yielded a log-likelihood value of the data. Other parameters were chosen as followed: admixed origin of populations was assumed and allele frequencies were allowed to be independent; burn-in was set at 50,000 and the number of MCMC replication after burn-in were 300,000 times, as described by Li et al. [10].

## Results

### mtDNA: sequence variation

A partial sequence of 16S was sequenced for 393 individuals. We detected 139 polymorphic sites of which 128 were parsimony informative in the 483 bp fragments, while the other 11 sites were singleton variable sites. Nucleotide frequencies for this gene were 0.368, 0.122, 0.334 and 0.176 for T, C, A and G, respectively. With DnaSP v5.10, we divided 393 individuals into 33 haplotypes, with a haplotype diversity of 0.436 (standard deviation is 0.029), and variance of *Hd* is only 0.001. Most individuals assembled into two haplotypes (Hap2 and Hap5), and the most common haplotype (Hap2) has 288 individuals, equal to 73.28% of all the samples. For all individuals, the average number of polymorphic sites (S) and haplotype diversity (*Pi*) were 122 and 0.06, respectively (Table 1). In geographical populations, the S value found in Mengla (68), Menglun (44) were much higher than others (Table 1). And the Pi value in Menglun (0.425) is also remarkable (Table 1).

Based on geographical distance, we assembled 25 geographical populations into 20 groups, in which Changsha-Taojiang-Taoyuan, Guilin-Huarong, Menglun-Mengla, Quanzhou-Shuangpai were combined into one group,respectively. Genetic variation attributed to the genetic differences among *C. kiangsui* geographical groups is 0.140 in AMOVA (p = 0.000) (Table 2). And negative values of F_ST_ values are really common between different geographical populations (see Table S1). In the AMOVA analysis, the lowest population specific *F*_ST_ index (0.112) is in the Taojiang group (0.157) (Table 3). The test of neutrality based on 1000 simulating samplings was significantly negative, with a Tajima's D value of −2.53, p<0.01 and a Fu's Fs value of −15.447, p<0.1. The others indices, Sum of Squared deviation (SSD) = 0.0387, P<0.1 and Harpending's Raggedness index (HRI) = 0.083, p>0.1. As τ is 10.769 in the mismatch analysis and the index u is 2.3%, we get a population expansion time of 0.242 Ma.

**Table 2:** Analysis of molecular variance (AMOVA) of C. kiangsu based on geographical distances.

Source of variation	Df	Sum of squares	Variance components	Percentage of variation	φ-statistics	P value
**Among groups**	19	21.229	0.031 Va	13.98	φ_CT_ = 0.140	0.000
**Among population within groups**	5	2.855	0.023 Vb	10.42	φ_SC_ = 0.121	0.022
**Within populations**	368	62.643	0.170 Vc	75.59	φ_ST_ = 0.244	0.152
**Total**	392	86.728	0.224			

**Table 3:** Population-specific *F_ST_* indices.

Population	16S rRNA
**Changning**	0.224
**Changsha**	0.213
**Guangde**	0.225
**Guangning**	0.250
**Guilin**	0.290
**Hengyang**	0.290
**Huarong**	0.207
**Jianou**	0.250
**Jinping**	0.290
**Jinyunshan**	0.290
**Mayanghe**	0.290
**Mengla**	0.166
**Menglun**	0.164
**Nanjing**	0.290
**Quanzhou**	0.275
**Quzhou**	0.269
**Rongan**	0.273
**Shicheng**	0.290
**Shuangpai**	0.273
**Shucheng**	0.238
**Taojiang**	0.157
**Taoyuan**	0.203
**Wuhan**	0.249
**Zijinshan**	0.203
**Ziyang**	0.290

Assumed all group have a common ancestral population, these population-specific coefficients would represent the degree of evolution of particular populations from a common ancestral population.

### mtDNA data: phylogeography

**Figure 2:**
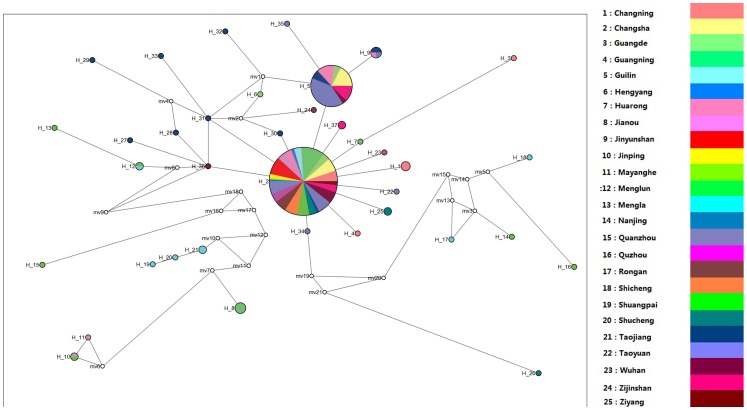
Most parsimonious median-joining (MJ) network for C. kiangsu 16S haplotypes. The size of the circles is proportional to the frequency of represented haplotypes. Geographical population indicated by colour as below. Nodes with small white circles are median vectors that represent hypothetical missing links or unsampled haplotypes.

The median-joining network (Figure 2) displays a star-like pattern, where the most common haplotype (Hap2) lies at the center and derivatives are connected to it. The most common haplotypes were found in all the sample sites except two geographical population (Menglun and Mengla), and the second most common haplotypes were found in seven sample sites. There are also several missing haplotypes on two main long branches.

The Neighbor-Joining tree appears to have an unclear topological structure (Figure S1). Two individuals from Jianou (Jianou_8 and Jianou_9) and one individual from Shuangpai (Shuangpai_17) joined in the branch belonging to the outgroup, C. nigricornis, and remained at the basement of the tree. This structure is supported by the MP tree. The individuals from Yunnan province were separated into two parts which conform to the actual geographical sampling location (Mengla and Menglun), although the distance between these two sample sites is only 90 km. The two Menglun individuals shared a branch with four individuals sampled from Guangning, while most Guangning individuals were placed in a huge branch with a high support level. Most sampled individuals belong to this large branch.

**Figure 3:**
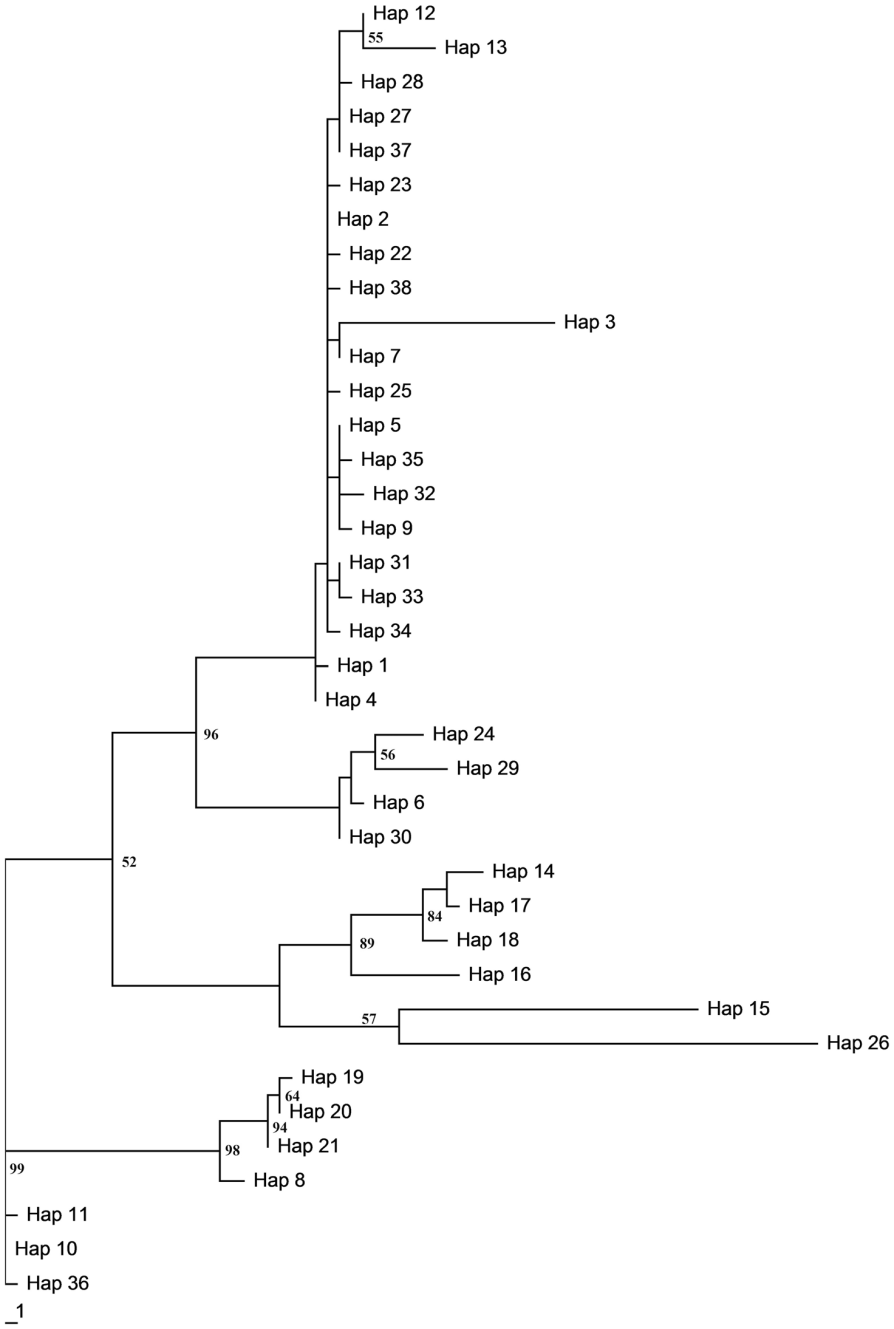
Maximum parsimony tree based on 16S rRNA haplotypes of 393 C. kiangsu individuals.

In the MP tree (Figure 3), with the exception of the three haplotypes from Jianou and Shuangpai, almost all haplotypes, including singletons, cluster together to form the main branch. The individuals from Yunnan are independent from the main branch and are separated from each other, at the same time sharing a branch with the same four Guangning individuals. The support for the MP tree is much less than that for the NJ tree, although they are unanimous in foundation structure.

### AFLP data: genetic structure and diversity

The four different primer combinations amplified 360 AFLP bands, of which the mean number of fragments per individual is 81.8. The number of segregating fragments is 310, about 86.1% of the total fragments. Guilin population has the most polymorphic loci (196) however Nanjign has the least polymorphic loci (11) (Table 4). Observed/effective number of alleles (*Na/Ne*) in each population is above 1.0 (Table 4). Nei's gene diversity (H) values in five populations are above 0.1, which are Guangning, Guilin, Huarong, Mengla and Shucheng (Table 4). The highest Nei's gene diversity is observed in Guangning (0.113), and the 2nd highest H is observed in Mengla (0.111) (Table 4). Shannon's Information indexes (*I*) were from 0.179 (Guangning) to 0.019 (Nanjing) (Table 4).In the population pairwise analysis, the F_ST_ indices between seven population pairs (Changsha – Menglun/Nanjing, Guilin – Menglun/Nanjing, Huarong – Menglun, Jinping – Mayanghe and Nanjing – Wuhan) are all 0, and the respective p values are all high (p>0.5), except Huarong – Menglun (*p* = 0.188) (see Table S2).

**Table 4:** The number of individuals sampled, observed number of alleles (*Na*), Effective number of alleles [Kimura and Crow (1964)](*Ne*), Nei's (1973) gene diversity (*H*), Shannon's Information index [Lewontin (1972)](*I*) and standard deviation of all index (St. Dev), the number of polymorphic loci in each population, and the percentage of polymorphic loci.

	*n*	*Na*	*St.Dev*	*Ne*	*St.Dev*	*H*	*St.Dev*	*I*	*St.Dev*	*loci*	percentage
**Changning**	10	1.386	0.488	1.129	0.230	0.087	0.136	0.144	0.207	139	38.61%
**Changsha**	10	1.456	0.499	1.126	0.192	0.091	0.124	0.155	0.195	164	45.56%
**Guangde**	9	1.264	0.441	1.113	0.245	0.072	0.140	0.113	0.209	95	26.39%
**Guangning**	10	1.419	0.494	1.175	0.276	0.113	0.159	0.179	0.236	151	41.94%
**Guilin**	10	1.544	0.499	1.137	0.197	0.100	0.121	0.174	0.189	196	54.44%
**Hengyang**	4	1.143	0.360	1.070	0.195	0.047	0.117	0.074	0.179	55	15.28%
**Huarong**	10	1.494	0.501	1.147	0.230	0.102	0.136	0.171	0.207	178	49.44%
**Jianou**	10	1.103	0.304	1.040	0.152	0.026	0.089	0.041	0.135	37	10.28%
**Jinping**	6	1.322	0.468	1.125	0.233	0.083	0.139	0.134	0.211	116	32.22%
**Jinyunshan**	10	1.381	0.486	1.135	0.234	0.091	0.140	0.149	0.213	137	38.06%
**Mayanghe**	2	1.078	0.268	1.055	0.190	0.032	0.111	0.047	0.162	28	7.78%
**Mengla**	3	1.319	0.467	1.181	0.294	0.111	0.170	0.169	0.253	115	31.94%
**Menglun**	2	1.128	0.334	1.090	0.236	0.053	0.139	0.077	0.202	46	12.78%
**Nanjing**	2	1.031	0.172	1.022	0.122	0.013	0.071	0.019	0.104	11	3.06%
**Quanzhou**	10	1.378	0.486	1.140	0.256	0.091	0.145	0.147	0.217	136	37.78%
**Quzhou**	10	1.200	0.401	1.066	0.183	0.044	0.108	0.072	0.164	82	20.00%
**Rongan**	10	1.389	0.488	1.127	0.228	0.086	0.135	0.143	0.205	140	38.89%
**Shicheng**	10	1.239	0.427	1.081	0.191	0.055	0.116	0.091	0.179	86	23.89%
**Shuangpai**	10	1.392	0.489	1.104	0.193	0.074	0.118	0.127	0.184	141	39.17%
**Shucheng**	10	1.394	0.489	1.159	0.271	0.101	0.154	0.162	0.229	142	39.44%
**Taojiang**	10	1.269	0.444	1.077	0.180	0.054	0.110	0.091	0.171	97	26.94%
**Taoyuan**	10	1.297	0.458	1.099	0.204	0.068	0.124	0.111	0.192	107	29.72%
**Wuhan**	10	1.350	0.478	1.079	0.144	0.061	0.099	0.108	0.163	126	35.00%
**Zijinshan**	10	1.442	0.487	1.129	0.221	0.009	0.130	0.152	0.199	159	44.17%
**Ziyang**	5	1.319	0.467	1.138	0.231	0.089	0.146	0.142	0.221	115	31.94%

**Figure 4:**
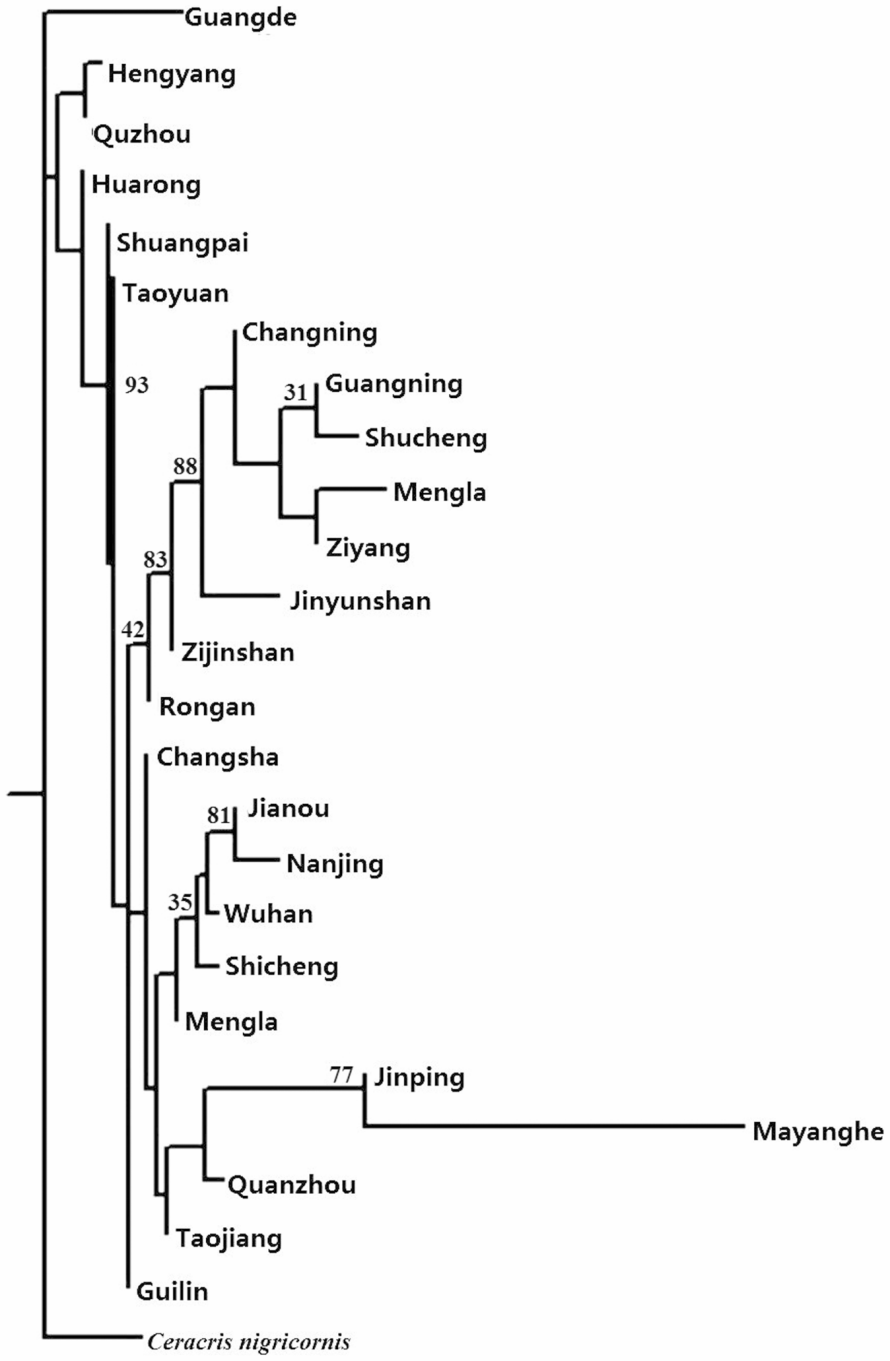
Arbitrarily rooted neighbour-joining tree based on Nei's genetic distances of AFLPs between 25 geographic populations.

In the neighbor-joining tree based on Nei's genetic distance shown in the Table S3, all individuals form three main branches (Figure 4). The Guilin, Rongan and Taoyuan groups remain at the center of the whole tree, while the Nanjing, Guangning and Guangde groups are the extremities of the three branches, respectively. The individuals of *C. nigricornis*, the outgroup, are not separated from *C. kiangsu* individuals but instead share the same branch with Guangde (Figure 4).

**Figure 5:**
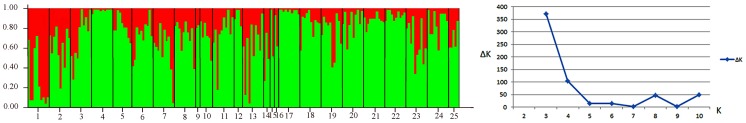
(a) Bayesian assignment proportions for K = 2 clusters determined in STRUCTURE 2.2 software. Each vertical bar represents one geographic populations; colours distinguish membership to different clusters. The number of each population are list as below: 1 = Shucheng; 2 = Quanzhou; 3 = Changning; 4 = Jianou; 5 = Guangde; 6 = Guilin; 7 = Guangning; 8 = Jinyunshan; 9 = Mayanghe; 10 = Jingpin; 11 = Huarong; 12 = Hengyang; 13 = Shicheng; 14 = Menglun; 15 = Mengla; 16 = Nanjing; 17 = Quzhou; 18 = Rongan; 19 = Changning; 20 = Shuangpai; 21 = Taojiang; 22 = Taoyuan; 23 = Wuhan; 24 = Zijinshan; 25 = Ziyang. (b) K (Evanno et al. 2005) as a function of K. K calculations not possible for K = 1 or the maximum consecutive value of K tested (K = 10).

For the purpose of testing for possible gene introgression among different populations, we used a Structure analysis based on a Bayesian model to infer the population admixture of the *C. nigricornis* populations (Figure 5a). Testing K (the number of distinct groups ranging from 2 to 16) only the Jianou and Quzhou populations are in a monophyletic group (K = 2 to 4), while the other populations show a farraginous mass of population structure (Figure 5b).

## Discussion

### Population structure

Our study, despite some contradictions between AFLP and mitochondrial DNA, reveals that there is a high level of genetic diversity and a low genetic distance among the individuals in the *C. kiangsu* population as a whole. The disparity of the genetic structure values in AMOVA may be due to differences in the base substitution rate in the nuclear data (AFLP) and the mitochondrial gene (*16S*). Furthermore, the variation tendency in both AMOVA and mismatch analysis were consistent with each other. The phylogenetic analysis highly supported monophyly of *C. kiangsui* (bootstrap value = 1000). However, when using AFLP and *16S* as markers, there is no clear phylogeographic structure in either the neighbor-joining or the maximum parsimony tree. Instead, some populations from distant geographic areas appeared on the same branch. Despite the possibility of some basic structure in the NJ and MP trees, there is currently little support for the branches in either tree. The results in our study, taken together, support the hypothesis that the *C. kiangsu* population is in a primary differentiation stage.

Jiang et al. (2011) amplified 506 bp of *16S* mtDNA from 5 populations of *Rammeacris kiangsu* Tsai (i.e. *C. kiangsu*). Four of their sampling locations were the same as ours, with the exception of Yongchang, which is not far from Jinyun Mountain. Their findings support our conclusion that no apparent population differentiations exist among these five geographical populations. Their NJ tree was constructed using 5 populations and *O. chinensis* as outgroup. Only the populations in Changning and Taojiang had some divergence from each other [61]. However, the genetic distance in their study was only 0.004, while our pairwise distance calculated in MEGA5 between Taojiang and Changning is 0.008. This difference between these two studies may be due to the different sampling number. We also found that C. nigricornis is not a suitable outgroup candidate due to the two C. nigricornis were not separated well from *C. kiangsu*.

There are several possible factors responsible for genetic differentiation of the populations in phytophagous insects, from low dispersal ability and geographical barriers, to habitat fragmentation and host plant availability [62]. Generally, geographic distance plays an important role in gene flow and diversity. But migratory species are often deemed to have less phylogeographic structure in their distribution range, as strong gene flow effects the homogenization of genetic variation as well as counteracts random drift, selection and mutation [63]–[66]. Higherφwithin populations than among populations suggests higher level of gene flow, in the light of the high dispersal ability reported for *C. kiangsu*, high levels of gene flow among populations and orderless phylogeographic relationship are perhaps convincing [67]. However, it has been shown that on large spatial scales, the ability of migration may not fully counteract the effect of geographical isolation. Therefore, geographic isolation might still be the preeminent factor in the population differentiation of the locust, while on a smaller spatial scale, the effects of geographical isolation might decrease and the effects of migration might increase [68]. But in our study, although our sampling range covered the all range of *C. kiangsu* from east to southeast of China. Most of our samples were assembled and clustered into one big clade. Weak differentiation signs can be found in the whole *C. kiangsu* population. Besides, the result of STRUCTURE (Fig. 5) shows poorer regularity than previous studies [10]. It means although *C. kiangsu* may have experienced population declines, the following events may be more important, like human activity which creates strong signal of introgression between different geographical populations.

It is important to note that there are potential limitations in using AFLPs for phylogenetic reconstruction [69], [70], some of which are due to the sharing of absences present in the calculation of distances [34]. The degree of homoplasy in the data and suitable distance measures for tree-building also seem to be important in this process [71]. Mitochondrial DNA is a marker among the most frequently used in analyses due to its maternal inheritance, haploid status, high rate of evolution and practicability [72]–[77], [2]. However, mtDNA may not be ideal for characterizing populations with a high frequency of a common haplotype [78]. Our study may have too small of a sampling number - for different geographical populations' shared same haplotypes the individuals from some locations are less than 10. Thus many local haplotypes may not have been included in our analyses. And the abnormal condition of Mayanghe population shown in AFLP analysis (far genetic distance and long NJ branch) were also attributed to the small sampling size. Intensive sampling must be employed in our future work. Furthermore, the incomplete nuclear lineage sorting or gene flow [79] may be responsible for the lack of AFLP structure between well supported mtDNA results [80], like the position of the Guilin population. Because the collection number of specimens is too low at several points, it is hard to determine the degree of diversity at Menglun, Nanjing and Mayanghe. We plan on continuing to increase the sample size and make use of 4-base selective primer pairs in AFLP analysis as part of our future work.

### Geographical isolation

The Hengduan Mountains have been reported as refugia for *O. hyla* intricata, and during the Quaternary, *O. hyla* intricata was distributed along the altitudinal gradient of the mountains. Following free retreats and expansions, frequent gene exchanges may have occurred among populations [10]. In our sampling range, Changning, Jinyunshan and Ziyang are near Hengduan Mountains and separated by Emei Mountain, so genetic distance among the locust groups from these locations were quite far. However, there are no mountains as high as the Hengduan Mountains in the southeast of China. Wuyi Mountains and Naling Mountains are the main mountains in the southeast of China, and their height above sea level is about 350 m and 1000 m, receptively, and prominent peaks only as high as 1,800 m and 2,200 m, respectively. But among some sampling locations around Wuyi Mountains and Nanling Mountains, like Jianou, Shicheng and Nanjing, which are located on both sides of Wuyi Mountains, F_ST_ between Shicheng and Nanjing and between Shicheng and Jianou is above 0.1. Wuyi Mountains may cut off the migration path between these locations. At the same time, gene flow likely exists between Taoyuan and Huarong, and the Qinlin Mountains and Daba Mountains do not obstruct the gene flow between the two populations. We took this phenomenon as the evidence of preliminary geographical isolation, although the whole population structure of *C. kiangsu* is still vague. However higher variation were found within population than among geographical group (Table 2), suggesting that this geographical isolation is still weak.

### Effect of the Quaternary glaciations events

Species divergence induced by the effects of genetic drift has often been linked to divergence that took part during displacements into glacial refugia and recolonization of previously glaciated areas [15], and the Pleistocene glacial cycles have been shown to have played a direct role in species divergence [81]–[85], [15]. For example, *L. migratoria*, although it is a highly migratory species deriving from different ancestral refugial populations following postglacial expansions, form three groups within China: the Tibetan group, the South China group and the North China group [20]. Zhang et al. (2009) attributed high haplotype diversity in populations of a mountain frog *Leptobrachium ailaonicum* to climatic oscillations during glacial periods in the Quaternary which allowed for population expansions while others remained stable [17].

In view of Tajima's D and Fu's Fs identified in the 16S, which were surprisingly below zero [86], [87], a population explosion of *C. kiangsu* likely occurred between 0.242 Ma. This range is within the Quaternary, between Stage II and Stage III of Penultimate Glaciation [88]. Thus, the Quaternary glaciation events appear to be linked to population explosion. Most likely the *C. kiangsu* species migrated to one refugium where frequent gene flow occurred among different ancestral populations, followed by postglacial expansions. Because of a lack of geographical isolation, gene flow between the geographical populations may have been quite frequent. This also explains why there are so many haplotypes shared among different geographical populations and so many assemblages in one phylogeographic branch. As we assumed all *C. kiangsu* has a common ancestral population, Menglun population, Mengla population and Taojiang population have smallest degree of differentiation based on the population specific *F*_ST_ indices (Table 2). This suggests the populations in Menglun, Mengla and Taojiang were more primitive. Although Changning, Guangning, Taojiang, Taoyuan and Mengla all have a high degree of haplotype diversity, we consider Mengla of Yunnan province as the main refugium, due to the fewer sampling number and its high haplotype and nucleotide diversity (Table 1) as well as its phylogenetic placement in both the *16S* MP tree (Fig. 3) and AFLPs NJ tree (Fig. 4) [89], supporting the refugial region Yunnan proposed by Chen et al. [2].

### Host plants

Some parasite species may have host-based genetic structure, like *Nomuraea rileyi* [90], and some insects with a strong ability of migration also depend on their host plants [36]. This even holds true for some vertebrate animals like certain bird species [91]. As we mentioned above, there is a unique bamboo species, *B. textilis*, in Guangning. However, it is hard to say whether different kinds of host plants have real influence on genetic differences, because the individuals from Guangning are not different from other individuals in either the NJ or MP tree. Furthermore, identification of genetic structure depends on the marker system [92]. Our AMOVA analysis did not reveal any noticeable differences of mtDNA, *F_ST_* or *Nst* for the Guangning individuals when compared to other sites, while in the AFLPs, these same individuals have an outstanding percentage of polymorphic loci, observed number of alleles (*Na*), effective number of alleles (*Ne*), Nei's gene diversity (*H*) and Shannon's Information index (*I*). All of these index values were second only to those of the Mengla group. In addition, in this study, only one location (Guangning) has the bamboo species *B. textilis*, it is hard to explain the complicated genetic relationship within the Guangning population. In the light of the high migration ability of *C. kiangsu*, a difference in host plants is perhaps not one of the factors generating hereditary difference. However, in the purpose of further clarifying the problem, high density sample data aimed at Guangning and typical locations of other host plants is specially needed in the future.

### Human activity

Some insecticide treatments may lead to nichetargeting selected and modified the genetic structure of injurious insect [93]. In our sampling sites, some individuals were from nature parks or landscapes, like the populations in Mengla county and Purple Mountain of Nanjing, and some were from an artificial forest, as the population in Wuhan. Thus, we propose that the *C. kiangsu* individuals from these places had experience a recent selective pressures resulting from human activities and conduced genetic variation in population.

Human project often generates habitat fragmentation, and this can influence the ecology and genetic diversities of organisms. Habitat fragmentation can especially influence mimicry in species in an endangered habitat, for example *Heliconius* butterflies in Brazil's Atlantic Forest [94]. Highly fragmented distributions were considered to cause deep genetic structure and strong differentiation [95]. Some continuous human activities such as destruction of forest for reclamation, grazing, mine exploitation, and cutting of firewood, herbicide application and sometimes even certain types of afforestation play important roles in wild species population declines [95]–[99], [10]. Even some short-term and localized programms considered to be uninjurious could cause negatively affect in insects that are not very good at migration, like *Palingenia longicauda*, and decrease genetic diversity [100]. Grasshopper control in agricultural practices reduces the population size and genetic diversity in populations [101]. The consequences of population size reductions include particularly low variability and marked genetic signatures [95]. Although many species have suffered population declines, increased population fragmentation, or even extinction in connection with these human impacts, others seem to have benefitted from human modification of their habitat, such as an insectivorous bat (*Tadarida brasiliensis mexicana*) [102]. In our study, the close genetic distance and low number of haplotypes among the Hengyang, Shuangpai, Quanzhou and Guilin populations, which exist despite the high elevation of Xuefeng Mountain, is easily linked to a series of inter-provincial highways flowing along the area, as well as some large-scale project like South-to-north Water Diversion, Three Gorges Dam, etc. These projects reduced some geographical barriers and provided potential new migration path to C. kiangsu. But at the same time, human trade activities and plant breeding activities also cause a high genetic exchange of agricultural pest [103], even cause invasive alien species [104]. In 1960s, central government launched a great project of bamboo transplanting, called South Bamboo Transfer to North, and the purpose of this project is transplanting well-grown bamboo species into north appropriate areas to afforest north cities and construct ecological engineering. To date, there are many bamboo plants, primarily *P. pubescebns*, had been transplanted from southern China to northern. For example, Henan province import first batch of P. pubescebns from Guangxi province in 1966, and from 1968 to 1976, 34 cities of Henan province set up hundreds of small nurseries, and cultivated 16.3 hm^2^ of bamboo plants [105], [106]. Another example, from 1965 to 2003, 130 bamboo species of 17 category were transplanted into Heze county of Hubei province, total cultivated area was 100.05 hm^2^. Then, about 10000 bamboo individuals in Heze county were transplanted into Beijing, Taiyuan, Lanzhou, Wuhan (one of our sampling sites), and Chengdu cities as greening plants [107]. Transplanting in huge amounts also occurred in Dayu Scenic Bamboo Garden in Yangzhou of Jiangsu province from 1999 to 2011, there are 22.8 hm^2^ transplanted bamboo [108]. And several bamboo botanic gardens like that were also set up in other places like Huaan of Fujian province [109], and Yiyang of Hunan [110]. In consideration of *C. kiangsu* spawn into root soil of bamboo and bamboo species introduction and transplantation technical requirements, so large amounts of transplanting may spread the eggs of *C. kiangsu* from one geographical population to others. In these locations, it is hard to prevent extraneous haplotypes from being introduced. In consideration of the graph shown in *16S* results (Figure 2), human activity may be the most fundamental reason of the recent colonization and population expasion. And our future work will investigate the sources of the main host plant of *C. kiangsu* and the migration path of *C. kiangsu*.

## Conclusions

Generally, geographic distance and geographical barriers probably play a considerable role in structuring genetic variation within species, although some migratory species may have less phylogeographic structure on smaller spatial scales [111]. In this study, 393 individuals collected from South and Southeast China were markered with mitochondrial DNA (*16S rRNA*), and 203 individuals were examined using AFLPs. Our results indicate that there is a high level of genetic diversity and a low genetic distance among the C. kiangsu populations. Although the broad geographic range in this study covers a large part of the habitat of *C. kiangsu*, our study did not reveals a clear geography-related population structure in *C. kiangsu*.

According to our analyses, *C. kiangsu* should be considered a monophyletic group. All our results supported the hypothesis that *C. kiangsu* populations are in a primary divergent stage. There are several potential reasons for this result. The most likely factor is that there is little geographical isolation along most parts of our sampling range. Additionally, human activity, host plant selection and sexuality relativity factors also potentially affect the species' phylogeographic structure. More research is required to determine the strength of these three factors.

The Tajima's D and Fu's Fs values identified in the 16S rRNA, which were unexpectedly below zero, reveal that there was a population explosion during the species history of *C. kiangsu*, and our mismatch analysis indicated that the population explosion likely occurred about 0.242 Ma. The Quaternary glaciation events may have been responsible for this population explosion. According to the phylogenetic trees and haplotype distribution, Mengla of Yunnan province, Changning of Sichuan province and Guangning of Guangdong province are possible refugia of *C. kiangsu*. Furthermore, those populations colonizationed in Mengla may have been separated from the other two refugia after the Quaternary glaciation. Because of the subsequent lack of physical barriers in East and South China [19], the main group was then free to migrate to most parts of the distribution range of *C. kiangsu*. And in our study, we first discovered human activity may have a significant impact on *C. kiangsu* population structure, which may be the most fundamental reason of current geographical population pattern. Our future work may focus on finding ancestor of different geographical populations.

## Supporting Information

Figure S1 NJ phylogenetic tree based on 16S rRNA gene sequences of 25 geographic populations of *C. kiangsu*.
(TIF)

Table S1 Computing conventional F-Statistics from haplotype frequencies, population pairwise *F_ST_* values (below diagonal) and *p* values (above diagonal) based on mtDNA data.
(DOCX)

Table S2 Population pairwise *F_ST_* values (below diagonal) and *p* value (above diagonal) based on AFLP data calculate from Arlequin software.
(DOCX)

Table S3 Nei's genetic distance after Lynch & Milligan method calculate from AFLPsurv software.
(DOCX)
