# Correction: Population Explosion in the Yellow-Spined Bamboo Locust *Ceracris kiangsu* and Inferences for the Impact of Human Activity

**DOI:** 10.1371/journal.pone.0118110

**Published:** 2015-02-06

**Authors:** The PLOS ONE Staff

There are errors in the author list. Benjamin Blanchard and Qi-Xin He should not be listed as authors on this article. The correct author list is: Zhou Fan, Guo-Fang Jiang, Yu-Xiang Liu. The correct citation is: Fan Z, Jiang G-F, Liu Y-X (2014) Population Explosion in the Yellow-Spined Bamboo Locust *Ceracris kiangsu* and Inferences for the Impact of Human Activity. PLoS ONE 9(3): e89873. doi:10.1371/journal.pone.0089873
