# Supplementary figures and images for: Population Explosion in the Yellow-Spined Bamboo Locust Ceracris kiangsu and Inferences for the Impact of Human Activity
Source: PLoS One. 2014 Mar 6;9(3):e89873. doi: 10.1371/journal.pone.0089873 (PMC3946154; doi:10.1371/journal.pone.0089873)

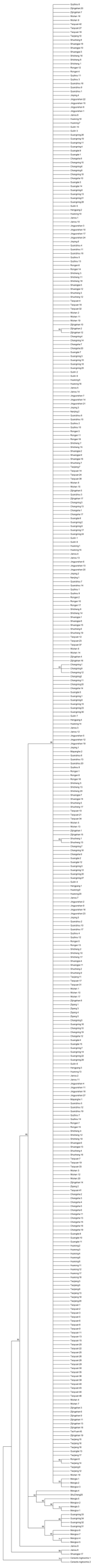

Supplement: Figure S1 — NJ phylogenetic tree based on 16S rRNA gene sequences of 25 geographic populations of C. kiangsu. (TIF) [file pone.0089873.s001.tif]
