# Supplementary material for: Population Explosion in the Yellow-Spined Bamboo Locust Ceracris kiangsu and Inferences for the Impact of Human Activity
Source: PLoS One. 2014 Mar 6;9(3):e89873. doi: 10.1371/journal.pone.0089873 (PMC3946154; doi:10.1371/journal.pone.0089873)
Supplement: Table S1 — Computing conventional F-Statistics from haplotype frequencies, population pairwise FST values (below diagonal) and p values (above diagonal) based on mtDNA data. (DOCX) [file pone.0089873.s002.docx]

**SUPPORTING INFORMATION**

**Table S1** Computing conventional *F*-Statistics from haplotype frequencies, population pairwise *F_ST_* values (below diagonal) and *p* values (above diagonal) based on mtDNA data.

|  | Changning | Changsha | Guangde | Guangning | Guilin | Hengyang | Huarong | Jianou | Jinyunshan | Jinping | Mayanghe | Mengla | Menglun | Nanjing | Quanzhou | Quzhou | Rongan | Shicheng | Shuangpai | Shucheng | Taojiang | Taoyuan | Wuhan | Zijinshan | Ziyang |
| --- | --- | --- | --- | --- | --- | --- | --- | --- | --- | --- | --- | --- | --- | --- | --- | --- | --- | --- | --- | --- | --- | --- | --- | --- | --- |
| Changning | * | 0.004 | 0.251 | 0.106 | 0.159 | 0.557 | 0.013 | 0.282 | 0.010 | 0.388 | 0.999 | 0.004 | 0.000 | 0.999 | 0.060 | 0.162 | 0.102 | 0.052 | 0.100 | 0.288 | 0.000 | 0.001 | 0.217 | 0.019 | 0.543 |
| Changsha | 0.250 | * | 0.096 | 0.000 | 0.006 | 0.114 | 0.636 | 0.004 | 0.000 | 0.050 | 0.466 | 0.006 | 0.003 | 0.440 | 0.000 | 0.004 | 0.000 | 0.000 | 0.001 | 0.003 | 0.066 | 0.999 | 0.010 | 0.445 | 0.044 |
| Guangde | 0.023 | 0.108 | * | 0.089 | 0.160 | 0.521 | 0.264 | 0.287 | 0.011 | 0.444 | 0.999 | 0.010 | 0.001 | 0.999 | 0.037 | 0.156 | 0.088 | 0.029 | 0.082 | 0.329 | 0.009 | 0.048 | 0.480 | 0.303 | 0.522 |
| Guangning | 0.045 | 0.362 | 0.063 | * | 0.300 | 0.999 | 0.000 | 0.344 | 0.096 | 0.688 | 0.999 | 0.001 | 0.000 | 0.999 | 0.236 | 0.444 | 0.298 | 0.163 | 0.289 | 0.202 | 0.000 | 0.000 | 0.279 | 0.000 | 0.710 |
| Guilin | 0.082 | 0.416 | 0.114 | 0.022 | * | 0.999 | 0.013 | 0.493 | 0.999 | 0.999 | 0.999 | 0.001 | 0.000 | 0.999 | 0.999 | 0.999 | 0.999 | 0.999 | 0.999 | 0.354 | 0.000 | 0.001 | 0.540 | 0.026 | 0.999 |
| Hengyang | -0.023 | 0.316 | -0.002 | -0.070 | 0.000 | * | 0.219 | 0.999 | 0.999 | 0.999 | 0.999 | 0.011 | 0.003 | 0.999 | 0.999 | 0.999 | 0.999 | 0.999 | 0.999 | 0.718 | 0.013 | 0.076 | 0.999 | 0.224 | 0.999 |
| Huarong | 0.147 | -0.030 | 0.020 | 0.242 | 0.287 | 0.184 | * | 0.023 | 0.000 | 0.096 | 0.551 | 0.021 | 0.003 | 0.517 | 0.002 | 0.011 | 0.003 | 0.001 | 0.004 | 0.013 | 0.069 | 0.506 | 0.048 | 0.843 | 0.114 |
| Jianou | 0.011 | 0.312 | 0.026 | -0.004 | 0.009 | -0.101 | 0.193 | * | 0.130 | 0.999 | 0.999 | 0.002 | 0.000 | 0.999 | 0.556 | 0.999 | 0.571 | 0.164 | 0.713 | 0.585 | 0.000 | 0.000 | 0.794 | 0.026 | 0.999 |
| Jinyunshan | 0.175 | 0.542 | 0.229 | 0.077 | 0.000 | 0.000 | 0.408 | 0.093 | * | 0.999 | 0.999 | 0.000 | 0.000 | 0.999 | 0.457 | 0.339 | 0.422 | 0.999 | 0.418 | 0.047 | 0.000 | 0.000 | 0.078 | 0.000 | 0.999 |
| Jinping | 0.031 | 0.360 | 0.055 | -0.020 | 0.000 | 0.000 | 0.231 | -0.044 | 0.000 | * | 0.999 | 0.002 | 0.001 | 0.999 | 0.999 | 0.999 | 0.999 | 0.999 | 0.999 | 0.519 | 0.001 | 0.015 | 0.708 | 0.088 | 0.999 |
| Mayanghe | -0.190 | 0.218 | -0.170 | -0.241 | 0.000 | 0.000 | 0.062 | -0.292 | 0.000 | 0.000 | * | 0.040 | 0.059 | 0.999 | 0.999 | 0.999 | 0.999 | 0.999 | 0.999 | 0.999 | 0.111 | 0.229 | 0.999 | 0.615 | 0.999 |
| Mengla | 0.366 | 0.309 | 0.356 | 0.556 | 0.643 | 0.452 | 0.283 | 0.481 | 0.803 | 0.538 | 0.286 | * | 0.703 | 0.052 | 0.000 | 0.000 | 0.000 | 0.000 | 0.000 | 0.002 | 0.128 | 0.010 | 0.000 | 0.011 | 0.007 |
| Menglun | 0.359 | 0.307 | 0.348 | 0.537 | 0.589 | 0.424 | 0.284 | 0.457 | 0.751 | 0.496 | 0.288 | -0.003 | * | 0.055 | 0.000 | 0.000 | 0.000 | 0.000 | 0.000 | 0.000 | 0.021 | 0.001 | 0.000 | 0.002 | 0.003 |
| Nanjing | -0.190 | 0.218 | -0.170 | -0.241 | 0.000 | 0.000 | 0.062 | -0.292 | 0.000 | 0.000 | 0.000 | 0.286 | 0.288 | * | 0.999 | 0.999 | 0.999 | 0.999 | 0.999 | 0.999 | 0.118 | 0.217 | 0.999 | 0.602 | 0.999 |
| Quanzhou | 0.086 | 0.436 | 0.116 | 0.025 | -0.040 | -0.138 | 0.306 | 0.002 | 0.011 | -0.083 | -0.331 | 0.670 | 0.628 | -0.331 | * | 0.999 | 0.999 | 0.999 | 0.999 | 0.212 | 0.000 | 0.000 | 0.327 | 0.002 | 0.999 |
| Quzhou | 0.048 | 0.378 | 0.071 | 0.005 | -0.026 | -0.132 | 0.251 | -0.023 | 0.044 | -0.075 | -0.328 | 0.586 | 0.548 | -0.328 | -0.027 | * | 0.999 | 0.392 | 0.999 | 0.364 | 0.000 | 0.000 | 0.517 | 0.015 | 0.999 |
| Rongan | 0.074 | 0.418 | 0.102 | 0.019 | -0.037 | -0.136 | 0.289 | -0.006 | 0.019 | -0.081 | -0.330 | 0.646 | 0.605 | -0.330 | -0.027 | -0.031 | * | 0.455 | 0.999 | 0.211 | 0.000 | 0.001 | 0.387 | 0.003 | 0.999 |
| Shicheng | 0.144 | 0.500 | 0.191 | 0.061 | 0.000 | 0.000 | 0.367 | 0.066 | 0.000 | 0.000 | 0.000 | 0.760 | 0.705 | 0.000 | -0.003 | 0.023 | 0.003 | * | 0.462 | 0.073 | 0.000 | 0.000 | 0.228 | 0.000 | 0.999 |
| Shuangpai | 0.074 | 0.418 | 0.102 | 0.019 | -0.037 | -0.136 | 0.289 | -0.029 | 0.019 | -0.081 | -0.330 | 0.646 | 0.605 | -0.330 | -0.027 | -0.031 | -0.029 | 0.003 | * | 0.202 | 0.000 | 0.000 | 0.351 | 0.005 | 0.999 |
| Shucheng | 0.009 | 0.279 | 0.021 | 0.019 | 0.055 | -0.057 | 0.167 | -0.013 | 0.153 | 0.000 | -0.236 | 0.419 | 0.405 | -0.236 | 0.051 | 0.016 | 0.040 | 0.120 | 0.040 | * | 0.002 | 0.001 | 0.457 | 0.021 | 0.693 |
| Taojiang | 0.214 | 0.079 | 0.158 | 0.357 | 0.370 | 0.256 | 0.069 | 0.280 | 0.508 | 0.307 | 0.141 | 0.049 | 0.067 | 0.141 | 0.409 | 0.344 | 0.390 | 0.463 | 0.390 | 0.244 | * | 0.040 | 0.000 | 0.052 | 0.000 |
| Taoyuan | 0.237 | -0.035 | 0.112 | 0.321 | 0.350 | 0.284 | -0.014 | 0.280 | 0.423 | 0.315 | 0.200 | 0.294 | 0.298 | 0.200 | 0.361 | 0.324 | 0.350 | 0.398 | 0.350 | 0.257 | 0.075 | * | 0.000 | 0.267 | 0.040 |
| Wuhan | 0.025 | 0.263 | -0.015 | 0.010 | 0.017 | -0.084 | 0.145 | -0.020 | 0.088 | -0.030 | -0.264 | 0.508 | 0.486 | -0.264 | 0.016 | -0.008 | 0.009 | 0.066 | 0.009 | 0.001 | 0.284 | 0.237 | * | 0.041 | 0.999 |
| Zijinshan | 0.121 | -0.010 | 0.008 | 0.213 | 0.254 | 0.150 | -0.038 | 0.163 | 0.372 | 0.198 | 0.021 | 0.261 | 0.264 | 0.021 | 0.273 | 0.219 | 0.256 | 0.332 | 0.256 | 0.138 | 0.070 | 0.005 | 0.122 | * | 0.111 |
| Ziyang | 0.009 | 0.341 | 0.032 | -0.040 | 0.000 | 0.000 | 0.211 | -0.067 | 0.000 | 0.000 | 0.000 | 0.500 | 0.464 | 0.000 | -0.105 | -0.098 | -0.103 | 0.000 | -0.103 | -0.023 | 0.285 | 0.302 | -0.052 | 0.178 | * |
