# Supplementary material for: Population Explosion in the Yellow-Spined Bamboo Locust Ceracris kiangsu and Inferences for the Impact of Human Activity
Source: PLoS One. 2014 Mar 6;9(3):e89873. doi: 10.1371/journal.pone.0089873 (PMC3946154; doi:10.1371/journal.pone.0089873)
Supplement: Table S2 — Population pairwise FST values (below diagonal) and p value (above diagonal) based on AFLP data calculate from Arlequin software. (DOCX) [file pone.0089873.s003.docx]

**Table S2** Population pairwise *Fst* values (below diagonal) and *p* value (above diagonal) based on AFLP data calculate from Arlequin software.

| Table 8 | ChangNing | ChangSha | GuangDe | GuangNing | GuiLin | HengYang | HuaRong | JianOu | JinPing | JinYunShan | MaYangHe | MengLa | MengLun | NanJing | QuanZhou | QuZhou | RongAn | ShiCheng | ShuangPai | ShuCheng | TaoJiang | TaoYuan | WuHan | ZiJinShan | ZiYang |
| --- | --- | --- | --- | --- | --- | --- | --- | --- | --- | --- | --- | --- | --- | --- | --- | --- | --- | --- | --- | --- | --- | --- | --- | --- | --- |
| ChangNing | * | 0.000 | 0.000 | 0.000 | 0.000 | 0.009 | 0.000 | 0.000 | 0.000 | 0.000 | 0.063 | 0.036 | 0.018 | 0.117 | 0.000 | 0.000 | 0.000 | 0.000 | 0.000 | 0.000 | 0.000 | 0.000 | 0.000 | 0.000 | 0.000 |
| ChangSha | 0.141 | * | 0.000 | 0.000 | 0.027 | 0.153 | 0.018 | 0.000 | 0.000 | 0.000 | 0.027 | 0.000 | 0.667 | 0.532 | 0.000 | 0.000 | 0.009 | 0.000 | 0.000 | 0.000 | 0.000 | 0.000 | 0.189 | 0.000 | 0.009 |
| GuangDe | 0.302 | 0.122 | * | 0.000 | 0.000 | 0.000 | 0.000 | 0.000 | 0.000 | 0.000 | 0.054 | 0.000 | 0.000 | 0.054 | 0.000 | 0.000 | 0.000 | 0.000 | 0.000 | 0.000 | 0.000 | 0.000 | 0.000 | 0.000 | 0.000 |
| GuangNing | 0.081 | 0.141 | 0.301 | * | 0.000 | 0.009 | 0.000 | 0.000 | 0.000 | 0.000 | 0.000 | 0.000 | 0.018 | 0.144 | 0.000 | 0.000 | 0.000 | 0.000 | 0.000 | 0.000 | 0.000 | 0.000 | 0.000 | 0.000 | 0.018 |
| GuiLin | 0.096 | 0.060 | 0.161 | 0.176 | * | 0.027 | 0.000 | 0.000 | 0.000 | 0.000 | 0.063 | 0.000 | 0.622 | 0.595 | 0.000 | 0.000 | 0.000 | 0.000 | 0.027 | 0.000 | 0.000 | 0.018 | 0.000 | 0.018 | 0.009 |
| HengYang | 0.201 | 0.038 | 0.183 | 0.197 | 0.082 | * | 0.270 | 0.000 | 0.009 | 0.000 | 0.090 | 0.018 | 0.126 | 0.252 | 0.000 | 0.018 | 0.036 | 0.000 | 0.009 | 0.000 | 0.009 | 0.045 | 0.009 | 0.045 | 0.009 |
| HuaRong | 0.134 | 0.045 | 0.150 | 0.157 | 0.049 | 0.023 | * | 0.000 | 0.000 | 0.000 | 0.189 | 0.018 | 0.162 | 0.225 | 0.000 | 0.000 | 0.054 | 0.000 | 0.135 | 0.000 | 0.000 | 0.027 | 0.000 | 0.027 | 0.000 |
| JianOu | 0.254 | 0.121 | 0.309 | 0.282 | 0.177 | 0.273 | 0.182 | * | 0.000 | 0.000 | 0.000 | 0.009 | 0.018 | 0.099 | 0.000 | 0.000 | 0.000 | 0.000 | 0.000 | 0.000 | 0.000 | 0.000 | 0.000 | 0.000 | 0.000 |
| JinPing | 0.143 | 0.093 | 0.238 | 0.200 | 0.085 | 0.197 | 0.101 | 0.272 | * | 0.000 | 0.532 | 0.018 | 0.342 | 0.279 | 0.000 | 0.000 | 0.000 | 0.000 | 0.000 | 0.000 | 0.009 | 0.000 | 0.000 | 0.000 | 0.000 |
| JinYunShan | 0.108 | 0.133 | 0.282 | 0.139 | 0.119 | 0.188 | 0.117 | 0.303 | 0.115 | * | 0.009 | 0.000 | 0.072 | 0.018 | 0.000 | 0.000 | 0.000 | 0.000 | 0.000 | 0.000 | 0.000 | 0.000 | 0.000 | 0.000 | 0.000 |
| MaYangHe | 0.208 | 0.134 | 0.373 | 0.226 | 0.106 | 0.402 | 0.138 | 0.531 | -0.001 | 0.169 | * | 0.108 | 0.342 | 0.324 | 0.045 | 0.009 | 0.045 | 0.027 | 0.000 | 0.009 | 0.018 | 0.018 | 0.036 | 0.063 | 0.036 |
| MengLa | 0.138 | 0.179 | 0.341 | 0.099 | 0.164 | 0.290 | 0.207 | 0.503 | 0.233 | 0.192 | 0.245 | * | 0.306 | 0.180 | 0.000 | 0.000 | 0.000 | 0.009 | 0.000 | 0.009 | 0.000 | 0.000 | 0.000 | 0.000 | 0.045 |
| MengLun | 0.146 | -0.022 | 0.228 | 0.194 | -0.016 | 0.155 | -0.001 | 0.389 | 0.056 | 0.130 | 0.253 | 0.149 | * | 0.360 | 0.117 | 0.036 | 0.261 | 0.261 | 0.423 | 0.027 | 0.117 | 0.117 | 0.216 | 0.171 | 0.099 |
| NanJing | 0.054 | -0.072 | 0.199 | 0.088 | -0.053 | 0.217 | 0.030 | 0.175 | 0.064 | 0.143 | 0.466 | 0.168 | 0.081 | * | 0.261 | 0.009 | 0.270 | 0.216 | 0.468 | 0.081 | 0.198 | 0.270 | 0.640 | 0.225 | 0.297 |
| QuanZhou | 0.122 | 0.106 | 0.290 | 0.116 | 0.121 | 0.174 | 0.088 | 0.259 | 0.087 | 0.117 | 0.170 | 0.253 | 0.124 | 0.072 | * | 0.000 | 0.000 | 0.000 | 0.000 | 0.000 | 0.000 | 0.000 | 0.000 | 0.000 | 0.000 |
| QuZhou | 0.223 | 0.099 | 0.219 | 0.248 | 0.137 | 0.095 | 0.117 | 0.161 | 0.269 | 0.244 | 0.468 | 0.386 | 0.247 | 0.185 | 0.231 | * | 0.000 | 0.000 | 0.009 | 0.000 | 0.000 | 0.009 | 0.000 | 0.000 | 0.000 |
| RongAn | 0.099 | 0.079 | 0.222 | 0.143 | 0.057 | 0.089 | 0.030 | 0.189 | 0.103 | 0.060 | 0.184 | 0.226 | 0.049 | 0.038 | 0.079 | 0.112 | * | 0.000 | 0.027 | 0.000 | 0.000 | 0.009 | 0.000 | 0.027 | 0.000 |
| ShiCheng | 0.187 | 0.136 | 0.336 | 0.264 | 0.150 | 0.233 | 0.174 | 0.204 | 0.131 | 0.214 | 0.269 | 0.342 | 0.057 | 0.042 | 0.173 | 0.250 | 0.148 | * | 0.000 | 0.000 | 0.000 | 0.000 | 0.000 | 0.000 | 0.000 |
| ShuangPai | 0.129 | 0.076 | 0.214 | 0.190 | 0.047 | 0.094 | 0.017 | 0.157 | 0.134 | 0.170 | 0.222 | 0.275 | 0.012 | 0.003 | 0.100 | 0.102 | 0.033 | 0.166 | * | 0.000 | 0.000 | 0.027 | 0.000 | 0.018 | 0.000 |
| ShuCheng | 0.111 | 0.163 | 0.311 | 0.080 | 0.181 | 0.253 | 0.192 | 0.314 | 0.218 | 0.201 | 0.261 | 0.164 | 0.228 | 0.116 | 0.178 | 0.283 | 0.192 | 0.291 | 0.222 | * | 0.000 | 0.000 | 0.000 | 0.000 | 0.000 |
| TaoJiang | 0.116 | 0.086 | 0.289 | 0.196 | 0.104 | 0.173 | 0.095 | 0.172 | 0.117 | 0.144 | 0.268 | 0.322 | 0.095 | 0.060 | 0.117 | 0.152 | 0.078 | 0.085 | 0.096 | 0.242 | * | 0.000 | 0.000 | 0.000 | 0.000 |
| TaoYuan | 0.130 | 0.087 | 0.175 | 0.195 | 0.058 | 0.123 | 0.052 | 0.182 | 0.147 | 0.160 | 0.276 | 0.283 | 0.128 | 0.026 | 0.136 | 0.107 | 0.049 | 0.225 | 0.043 | 0.223 | 0.133 | * | 0.000 | 0.009 | 0.000 |
| WuHan | 0.190 | 0.025 | 0.196 | 0.223 | 0.090 | 0.179 | 0.159 | 0.172 | 0.148 | 0.217 | 0.266 | 0.279 | 0.112 | -0.073 | 0.209 | 0.196 | 0.166 | 0.151 | 0.159 | 0.219 | 0.156 | 0.149 | * | 0.000 | 0.018 |
| ZiJinShan | 0.090 | 0.088 | 0.192 | 0.151 | 0.044 | 0.096 | 0.055 | 0.218 | 0.117 | 0.084 | 0.155 | 0.198 | 0.088 | 0.049 | 0.137 | 0.144 | 0.037 | 0.206 | 0.079 | 0.192 | 0.116 | 0.057 | 0.165 | * | 0.009 |
| ZiYang | 0.119 | 0.113 | 0.286 | 0.062 | 0.112 | 0.225 | 0.169 | 0.347 | 0.198 | 0.143 | 0.241 | 0.084 | 0.183 | 0.063 | 0.199 | 0.273 | 0.150 | 0.292 | 0.210 | 0.123 | 0.231 | 0.179 | 0.177 | 0.117 | * |
