# Supplementary material for: Population Explosion in the Yellow-Spined Bamboo Locust Ceracris kiangsu and Inferences for the Impact of Human Activity
Source: PLoS One. 2014 Mar 6;9(3):e89873. doi: 10.1371/journal.pone.0089873 (PMC3946154; doi:10.1371/journal.pone.0089873)
Supplement: Table S3 — Nei's genetic distance after Lynch & Milligan method calculate from AFLPsurv software. (DOCX) [file pone.0089873.s004.docx]

**Table S3** Nei’s genetic distance after Lynch & Milligan method calculate from AFLPsurv software.

| Table 6 | ChangNing | ChangSha | GuangDe | GuangNing | GuiLin | HengYang | HuaRong | JianOu | JinPing | JinYunShan | MaYangHe | MengLa | MengLun | NanJing | QuanZhou | QuZhou | RongAn | ShiCheng | ShuangPai | ShuCheng | TaoJiang | TaoYuan | WuHan | ZiJinShan | ZiYang |
| --- | --- | --- | --- | --- | --- | --- | --- | --- | --- | --- | --- | --- | --- | --- | --- | --- | --- | --- | --- | --- | --- | --- | --- | --- | --- |
| ChangNing | * |  |  |  |  |  |  |  |  |  |  |  |  |  |  |  |  |  |  |  |  |  |  |  |  |
| ChangSha | 0.005 | * |  |  |  |  |  |  |  |  |  |  |  |  |  |  |  |  |  |  |  |  |  |  |  |
| GuangDe | 0.022 | 0.006 | * |  |  |  |  |  |  |  |  |  |  |  |  |  |  |  |  |  |  |  |  |  |  |
| GuangNing | 0.001 | 0.007 | 0.025 | * |  |  |  |  |  |  |  |  |  |  |  |  |  |  |  |  |  |  |  |  |  |
| GuiLin | 0.002 | 0.000 | 0.010 | 0.011 | * |  |  |  |  |  |  |  |  |  |  |  |  |  |  |  |  |  |  |  |  |
| HengYang | 0.013 | 0.003 | 0.008 | 0.015 | 0.007 | * |  |  |  |  |  |  |  |  |  |  |  |  |  |  |  |  |  |  |  |
| HuaRong | 0.006 | 0.000 | 0.006 | 0.008 | 0.000 | 0.000 | * |  |  |  |  |  |  |  |  |  |  |  |  |  |  |  |  |  |  |
| JianOu | 0.009 | 0.002 | 0.012 | 0.012 | 0.006 | 0.006 | 0.005 | * |  |  |  |  |  |  |  |  |  |  |  |  |  |  |  |  |  |
| JinPing | 0.005 | 0.001 | 0.015 | 0.012 | 0.001 | 0.014 | 0.003 | 0.007 | * |  |  |  |  |  |  |  |  |  |  |  |  |  |  |  |  |
| JinYunShan | 0.004 | 0.008 | 0.022 | 0.008 | 0.007 | 0.015 | 0.007 | 0.017 | 0.004 | * |  |  |  |  |  |  |  |  |  |  |  |  |  |  |  |
| MaYangHe | 0.031 | 0.029 | 0.044 | 0.039 | 0.029 | 0.043 | 0.030 | 0.029 | 0.010 | 0.029 | * |  |  |  |  |  |  |  |  |  |  |  |  |  |  |
| MengLa | 0.004 | 0.009 | 0.024 | 0.004 | 0.010 | 0.020 | 0.015 | 0.021 | 0.015 | 0.012 | 0.045 | * |  |  |  |  |  |  |  |  |  |  |  |  |  |
| MengLun | 0.007 | 0.000 | 0.009 | 0.016 | 0.000 | 0.003 | 0.000 | 0.003 | 0.000 | 0.010 | 0.030 | 0.015 | * |  |  |  |  |  |  |  |  |  |  |  |  |
| NanJing | 0.010 | 0.004 | 0.017 | 0.014 | 0.007 | 0.013 | 0.010 | 0.002 | 0.010 | 0.020 | 0.036 | 0.023 | 0.003 | * |  |  |  |  |  |  |  |  |  |  |  |
| QuanZhou | 0.005 | 0.003 | 0.021 | 0.003 | 0.006 | 0.011 | 0.002 | 0.009 | 0.001 | 0.006 | 0.027 | 0.018 | 0.005 | 0.010 | * |  |  |  |  |  |  |  |  |  |  |
| QuZhou | 0.010 | 0.003 | 0.009 | 0.013 | 0.007 | 0.000 | 0.003 | 0.004 | 0.013 | 0.015 | 0.041 | 0.017 | 0.003 | 0.010 | 0.011 | * |  |  |  |  |  |  |  |  |  |
| RongAn | 0.002 | 0.000 | 0.012 | 0.006 | 0.000 | 0.004 | 0.000 | 0.005 | 0.002 | 0.001 | 0.030 | 0.013 | 0.000 | 0.009 | 0.001 | 0.002 | * |  |  |  |  |  |  |  |  |
| ShiCheng | 0.006 | 0.002 | 0.019 | 0.014 | 0.004 | 0.012 | 0.006 | 0.003 | 0.002 | 0.013 | 0.027 | 0.017 | 0.000 | 0.004 | 0.006 | 0.009 | 0.004 | * |  |  |  |  |  |  |  |
| ShuangPai | 0.006 | 0.001 | 0.011 | 0.010 | 0.000 | 0.003 | 0.000 | 0.004 | 0.006 | 0.011 | 0.033 | 0.018 | 0.000 | 0.006 | 0.003 | 0.002 | 0.000 | 0.006 | * |  |  |  |  |  |  |
| ShuCheng | 0.003 | 0.008 | 0.025 | 0.001 | 0.011 | 0.021 | 0.012 | 0.015 | 0.013 | 0.013 | 0.040 | 0.008 | 0.017 | 0.015 | 0.009 | 0.018 | 0.011 | 0.016 | 0.015 | * |  |  |  |  |  |
| TaoJiang | 0.003 | 0.000 | 0.015 | 0.009 | 0.002 | 0.007 | 0.001 | 0.003 | 0.001 | 0.007 | 0.027 | 0.014 | 0.000 | 0.005 | 0.003 | 0.004 | 0.000 | 0.000 | 0.001 | 0.013 | * |  |  |  |  |
| TaoYuan | 0.004 | 0.001 | 0.008 | 0.010 | 0.000 | 0.006 | 0.000 | 0.005 | 0.005 | 0.008 | 0.033 | 0.015 | 0.001 | 0.006 | 0.005 | 0.002 | 0.000 | 0.008 | 0.000 | 0.013 | 0.003 | * |  |  |  |
| WuHan | 0.008 | 0.000 | 0.010 | 0.013 | 0.000 | 0.011 | 0.006 | 0.003 | 0.003 | 0.014 | 0.033 | 0.013 | 0.000 | 0.002 | 0.010 | 0.009 | 0.006 | 0.002 | 0.007 | 0.011 | 0.003 | 0.005 | * |  |  |
| ZiJinShan | 0.002 | 0.002 | 0.011 | 0.009 | 0.000 | 0.005 | 0.000 | 0.009 | 0.004 | 0.002 | 0.028 | 0.011 | 0.002 | 0.012 | 0.007 | 0.006 | 0.000 | 0.009 | 0.001 | 0.012 | 0.002 | 0.000 | 0.007 | * |  |
| ZiYang | 0.003 | 0.002 | 0.018 | 0.001 | 0.003 | 0.014 | 0.009 | 0.010 | 0.008 | 0.006 | 0.036 | 0.000 | 0.010 | 0.010 | 0.011 | 0.011 | 0.005 | 0.012 | 0.011 | 0.004 | 0.008 | 0.006 | 0.004 | 0.004 | * |
